# Correction: Belisario et al. ABCA1/ABCB1 Ratio Determines Chemo- and Immune-Sensitivity in Human Osteosarcoma. *Cells* 2020, *9*, 647

**DOI:** 10.3390/cells14090622

**Published:** 2025-04-22

**Authors:** Dimas Carolina Belisario, Muhlis Akman, Martina Godel, Virginia Campani, Maria Pia Patrizio, Lorena Scotti, Claudia Maria Hattinger, Giuseppe De Rosa, Massimo Donadelli, Massimo Serra, Joanna Kopecka, Chiara Riganti

**Affiliations:** 1 Department of Oncology, University of Torino, Via Santena 5/bis, 10126 Torino, Italy; dimascarolina.belisario@unito.it (D.C.B.); muhlis.akman@unito.it (M.A.); martina.godel@edu.unito.it (M.G.); joanna.kopecka@unito.it (J.K.); 2 Department of Pharmacy, University of Napoli Federico II, Via D. Montesano 49, 80131 Napoli, Italy; virginia.campani@unina.it (V.C.); lorena.scotti@unina.it (L.S.); gderosa@unina.it (G.D.R.); 3 IRCCS Istituto Ortopedico Rizzoli, Laboratory of Experimental Oncology, Pharmacogenomics and Pharmacogenetics Research Unit, Via di Barbiano, 1/10, 40136 Bologna, Italy; mariapia.patrizio@ior.it (M.P.P.); claudia.hattinger@ior.it (C.M.H.); massimo.serra@ior.it (M.S.); 4 Department of Neurosciences, Biomedicine and Movement Sciences, Section of Biochemistry, University of Verona, Piazzale L.A. Scuro 10, 37134 Verona, Italy; massimo.donadelli@univr.it

## Error in Figures

In the original publication [1], there was a mistake in Figure 1B as published. During figure assembly, we left in two panels that belonged to the preparatory version of the publication because of a copy-and-paste clerical error. The corrected Figure 1 appears below.

In the original publication, there was a mistake in Figure 4A as published. The first blot of panel A was labelled as p(Thr202/Tyr204)ERK1/2, and the second blot of panel A was labelled as Total ERK1/2 because of a copy-and-paste clerical error. The first blot should be Total ERK1/2 and the second blot should be p(Thr202/Tyr204)ERK1/2, as it was correctly described in the text. The corrected Figure 4 appears below.

In the original publication, there was a mistake in Figure 5C as published. During figure assembly, we left two panels that belonged to the preparatory version of the publication because of a copy-and-paste clerical error. The corrected Figure 5 appears below.

The authors state that the scientific conclusions are unaffected. This correction was approved by the Academic Editor. The original publication has also been updated.

## Figures and Tables

**Figure 1:**
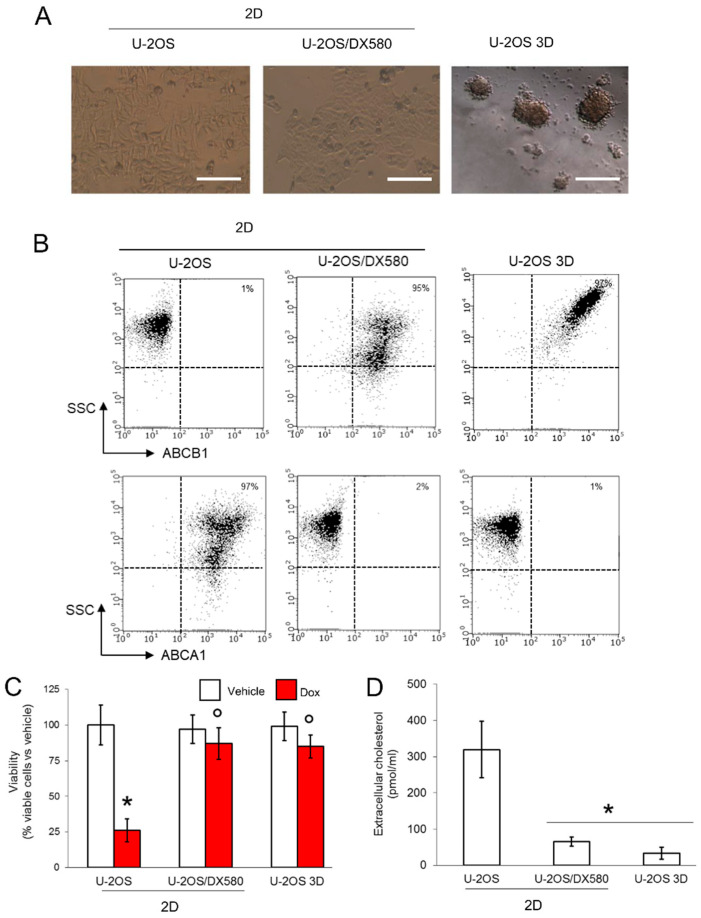
3D cultures of U-2OS cells display an ABCB1*^high^*ABCA1*^low^* phenotype. (**A**) Representative micro-photographs of U-2OS cells, U-2OS/DX580 cells, both grown as 2D cultures, and U-2OS cells grown in 3D culture (10× ocular lens, 4× objective). Bar: 100 μM. (**B**) Dot plots of ABCB1 and ABCA1 proteins on the cell surface, measured by flow cytometry in duplicates. The figure is representative of one out of three experiments. SSC: side scattering. Percentage of ABCB1- and ABCA1-positive cells, calculated as cells with a fluorescence >10 ^2^ using the Incyte software. (**C**) Cells were grown for 72 h in medium containing 5 μM DMSO (vehicle) or doxorubicin (Dox). Percentage of viable cells, measured by a chemiluminescence-based assay in quadruplicates. Data are means ± SD (n = 4 independent experiments). * *p* < 0.001 for doxorubicin-treated cells vs. untreated cells; ° *p* < 0.001 for doxorubicin-treated 2D U-2OS/DX580 and 3D U-2OS cells vs. doxorubicin-treated 2D U-2OS cells. (**D**) Cells were labeled 1 h with [^14^C]-cholesterol and extensively washed. After 24 h, the [^14^C]-cholesterol collected in the supernatant, considered an index of cholesterol efflux, was measured by liquid scintillation in duplicates. Data are means ± SD (n = 3 independent experiments). * *p* < 0.001 for 2D U-2OS/DX580 and 3D U-2OS cells vs. 2D U-2OS cells. ABCB1: ATP Binding Cassette transporter B1; ACBA1: ATP Binding Cassette transporter A1.

**Figure 4:**
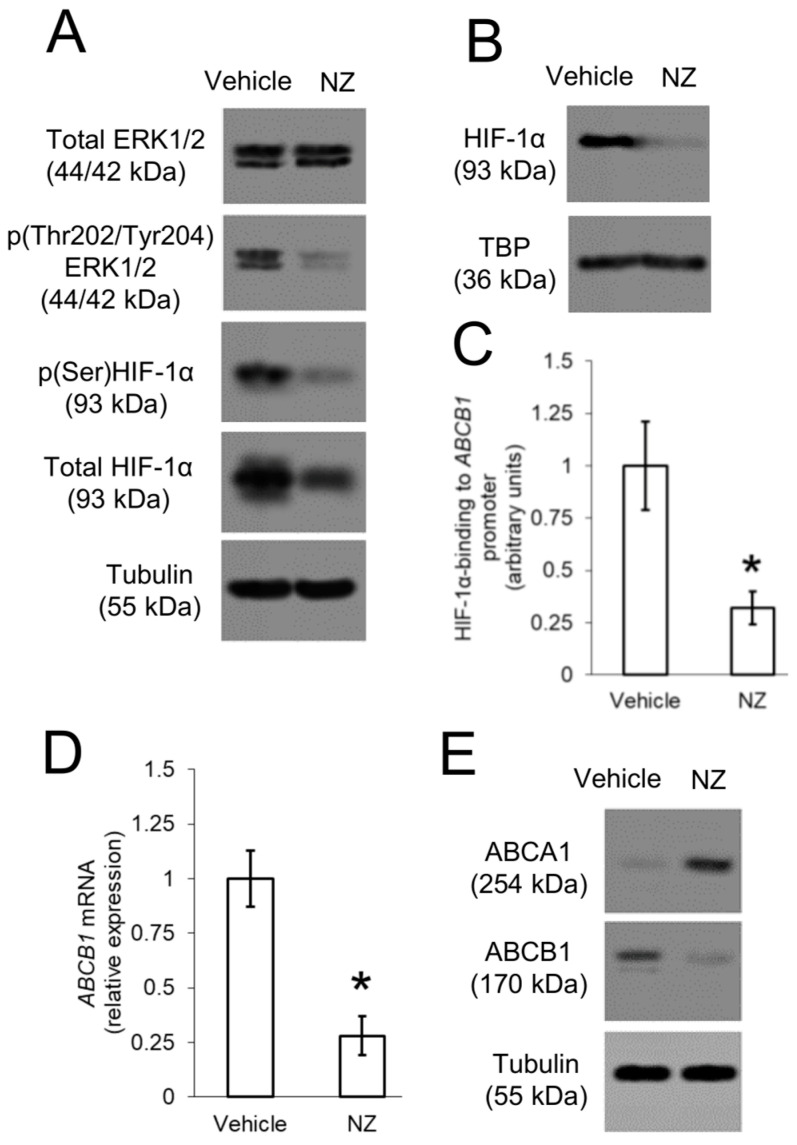
ABCB1 is up-regulated by ERK1/2/HIF-1α axis in resistant osteosarcoma cells. 3D U-2OS cells were treated with sterile physiological solution (vehicle) or self-assembled nanoparticles encapsulating zoledronic acid (NZ), containing 1 μM zoledronic acid for 24 h. (**A**) Immunoblotting of the indicated proteins in whole-cell extracts. The β-tubulin expression was used as control of equal protein loading. The figure is representative of one out of three experiments. (**B**) Immunoblotting of HIF-1α in nuclear extracts. The TBP expression was used as control of equal protein loading. The figure is representative of one out of three experiments. (**C**) HIF-1α binding to *ABCB1* promoter, measured by ChIP in triplicates. Data are means ± SD (n = 3 independent experiments). * *p* < 0.001 for NZ-treated cells vs. vehicle-treated cells. (**D**) ABCB1 mRNA levels, measured by qRT-PCR in triplicates. Data are means ± SD (n = 3 independent experiments). * *p* < 0.001 for NZ-treated cells vs. vehicle-treated cells. (**E**) Immunoblotting of the indicated proteins in whole-cell extracts. The β-tubulin expression was used as control of equal protein loading. The figure is representative of one out of three experiments.

**Figure 5:**
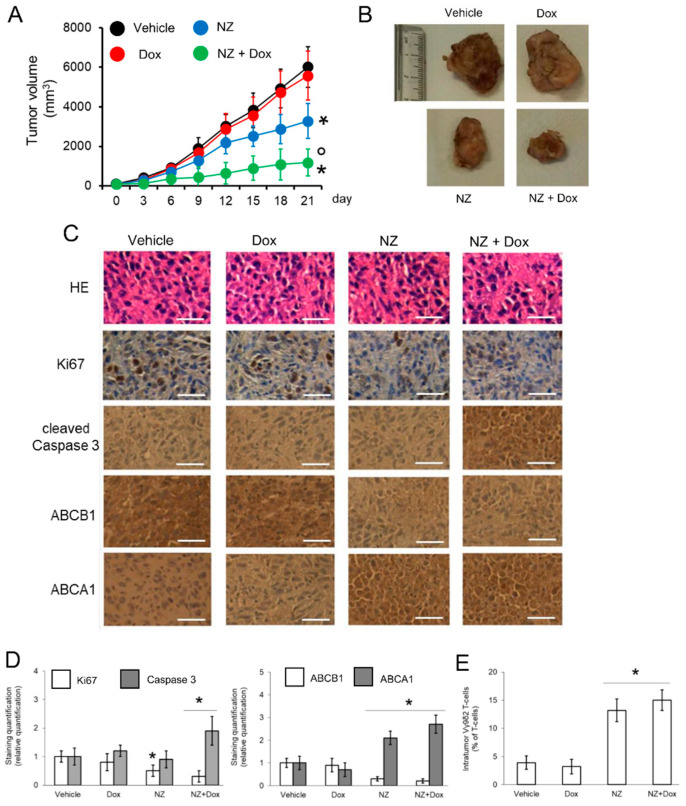
Self-assembled nanoparticles encapsulating zoledronic acid restores chemo-immune-sensitivity in vivo. U-2OS 3D cells (1 × 10^6^) were injected subcutaneously in Hu-CD34^+^ mice. When the tumor reached the volume of 50 mm^3^, animals (n = 8/group) were randomized and treated (on day 3, 9, and 15 after randomization) as it follows: 1) Vehicle group, treated with 0.1 ml saline solution intravenously (i.v.); 2) doxorubicin (Dox) group, treated with 5 mg/kg doxorubicin i.v.; 3) NZ group, treated with 20 μg/mice zoledronic acid i.v.; 4) NZ + Dox group, treated with 20 μg/mice zoledronic acid as NZ and 5 mg/kg doxorubicin i.v. (**A**) Tumor growth was monitored by caliper. * *p* < 0.01 for NZ-treated mice vs. vehicle-treated mice (days 15-21), * *p* < 0.001 for NZ + Dox-treated mice vs. vehicle-treated mice (days 9–21); ° *p* < 0.001 for NZ+Dox-treated mice vs. Dox-treated mice (days 9–21). (**B**) Representative photographs of tumors from each group of treatment. (**C**) Immunohistochemical analysis of tumor slices stained with hematoxylin-eosin (HE) or immunostained for the indicated proteins (63× objective). Bar = 10 μm. The microphotographs are representative of five tumors/each group. (**D**) The amount of Ki67, cleaved caspase 3, ABCB1, and ABCA1 positive cells were calculated using the Photoshop program. The staining intensity of the “Vehicle” group was considered 1. The staining intensity of the other groups was expressed as relative intensity staining vs. Vehicle group. * *p* < 0.01 for NZ-treated/NZ+Dox-treated cells vs. vehicle-treated cells. (**E**) Percentage of intratumor Vγ9δ2 T-lymphocytes vs. all CD3^+^T-lymphocyte-infiltrating cells, measured by flow cytometry. Data are means ± SD (n = 8 animals). * *p* < 0.001 for NZ-treated/NZ+Dox-treated cells vs. vehicle-treated cells.
