# Supplementary material for: ABCA1/ABCB1 Ratio Determines Chemo- and Immune-Sensitivity in Human Osteosarcoma
Source: Cells. 2020 Mar 6;9(3):647. doi: 10.3390/cells9030647 (PMC7140509; doi:10.3390/cells9030647)
Supplement: Supplementary file 1 [file cells-09-00647-s001.pdf]

ABCA1/ABCB1 Ratio Determines Chemo- and Immune-Sensitivity in Human Osteosarcoma

Dimas Carolina Belisario [1,†], Muhlis Akman [1,†], Martina Godel [1], Virginia Campani [2], Maria Pia Patrizio [3], Lorena Scotti [2], Claudia Maria Hattinger [3], Giuseppe De Rosa [2], Massimo Donadelli [4], Massimo Serra [3], Joanna Kopecka [1,‡] and Chiara Riganti [1,‡,*]

[1] Department of Oncology, University of Torino, via Santena 5/bis, 10126 Torino, Italy; dimascarolina.belisario@unito.it (D.C.B.); muhlis.akman@unito.it (M.A.); martina.godel@edu.unito.it (M.G.); joanna.kopecka@unito.it (J.K.)

[2] Department of Pharmacy, University of Napoli Federico II, Via D. Montesano 49, 80131, Napoli, Italy; virginia.campani@unina.it (V.C.); lorena.scotti@unina.it (L.S.); gderosa@unina.it (G.D.R.)

[3] IRCCS Istituto Ortopedico Rizzoli, Laboratory of Experimental Oncology, Pharmacogenomics and Pharmacogenetics Research Unit, via di Barbiano, 1/10, 40136, Bologna, Italy; mariapia.patrizio@ior.it (M.P.P.); claudia.hattinger@ior.it (C.M.H.); massimo.serra@ior.it (M.S.)

[4] Department of Neurosciences, Biomedicine and Movement Sciences, Section of Biochemistry, University of Verona, Piazzale L.A. Scuro 10, 37134, Verona, Italy; massimo.donadelli@univr.it

* Correspondence: chiara.riganti@unito.it; Tel.: +39-0116705857

† Equal contribution.

‡ Senior co-authorship.

Table S1: Expression of ABCB1 and ABCA1 genes in primary high-grade osteosarcoma tumor samples and in human normal tissues of reference

Figure S1: ABCB1 and ABCA1 expression and functions in Saos-2 cells

Figure S2: ABCB1 and ABCA1 mRNA expression and clinical outcome

Figure S3: IPP efflux and Vγ9δ2 T-lymphocytes activity against Saos-2 cells

Figure S4: Endogenous synthesis of cholesterol and IPP in osteosarcoma cells

Figure S5: Tumor weight of treated animals

Figure S6: Immunophenotype of the intratumor lymphocytic infiltrate

Table S1. Expression of ABCB1 and ABCA1 genes in primary high-grade osteosarcoma tumor samples and in human normal tissues of reference

**Gene Name (gene ID)**	***Probe identification***	**OS-IOR ITCC *(21 samples)***	**Normal Skeletal Muscle Asmann *(40 samples)***	**Normal Muscle Gordon *(22 samples)***
**ABCA1 (19)**	203504_s_at	365.90 *(range: 120.20 - 630.20)*	158.45 *(range: 104.60 - 272.40)*	241.10 *(range: 55.20 - 510.80)*
**ABCB1 (5243)**	243951_at	22.30 *(range: 8.50 - 46.05)*	16.75 *(range: 2.30 - 79.20)*	23.80 *(range: 3.60 - 50.70)*

The OS-IOR ITCC is a series of 21 primary, high-grade osteosarcomas of the extremities, non-metastatic at diagnosis, arisen in patients younger than 40 years collected at the Orthopaedic Rizzoli Institute (GEO ID: GSE87437), and shared within the Innovative Therapies for Children with Cancer European Consortium (ITCC; http://www.itcc-consortium.org/). The human normal tissues listed in the Table included the Asmann series of human normal skeletal muscle (GEO ID: GSE9103) and the Gordon series of human normal muscle (GEO ID: GSE38718) samples. Values listed in the table represent the median and range of gene expression level inside each dataset.

Figure S1

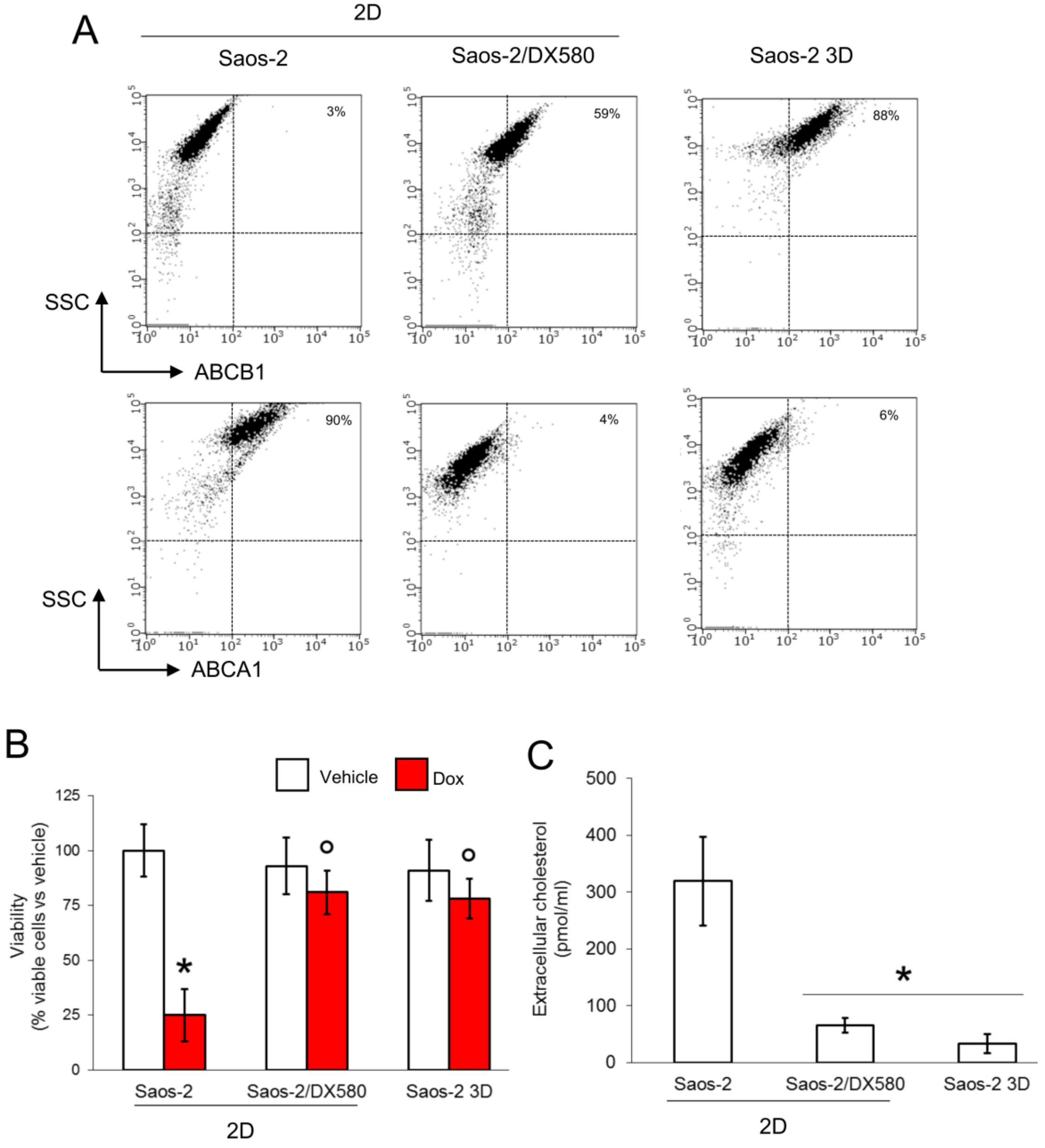

Figure S1. ABCB1 and ABCA1 expression and functions in Saos-2 cells

Saos-2 cells and Saos-2/DX580 cells were grown as 2D or 3D cultures. **A.** Dot plots of ABCB1 and ABCA1 proteins on cell surface, measured by flow cytometry in duplicates. The figure is representative of one out of three experiments. SSC: side scattering. Percentage of ABCB1- and ABCA1-positive cells, calculated as cells with a fluorescence > 10^{2} using the Incyte software. **B.** Cells were grown for 72 h in medium containing 5 μM DMSO (Vehicle) or doxorubicin (Dox). Percentage of viable cells, measured by a chemiluminescence-based assay in quadruplicates. Data are means±SD (n=3 independent experiments). *p<0.001 for doxorubicin-treated cells vs. untreated cells; °p<0.001 for doxorubicin-treated 2D Saos-2/DX580 and 3D Saos-2 cells vs. doxorubicin-treated 2D Saos-2 cells. **D.** Cells were labelled 1 h with [^{14}C]-cholesterol and extensively washed. After 24 h, the [^{14}C]-cholesterol collected in the supernatant, considered an index of cholesterol efflux, was measured by liquid scintillation in duplicates. Data are means±SD (n=3 independent experiments). *p<0.001 for 2D Saos-2/DX580 and 3D Saos-2 cells vs. 2D Saos-2 cells.

Figure S2

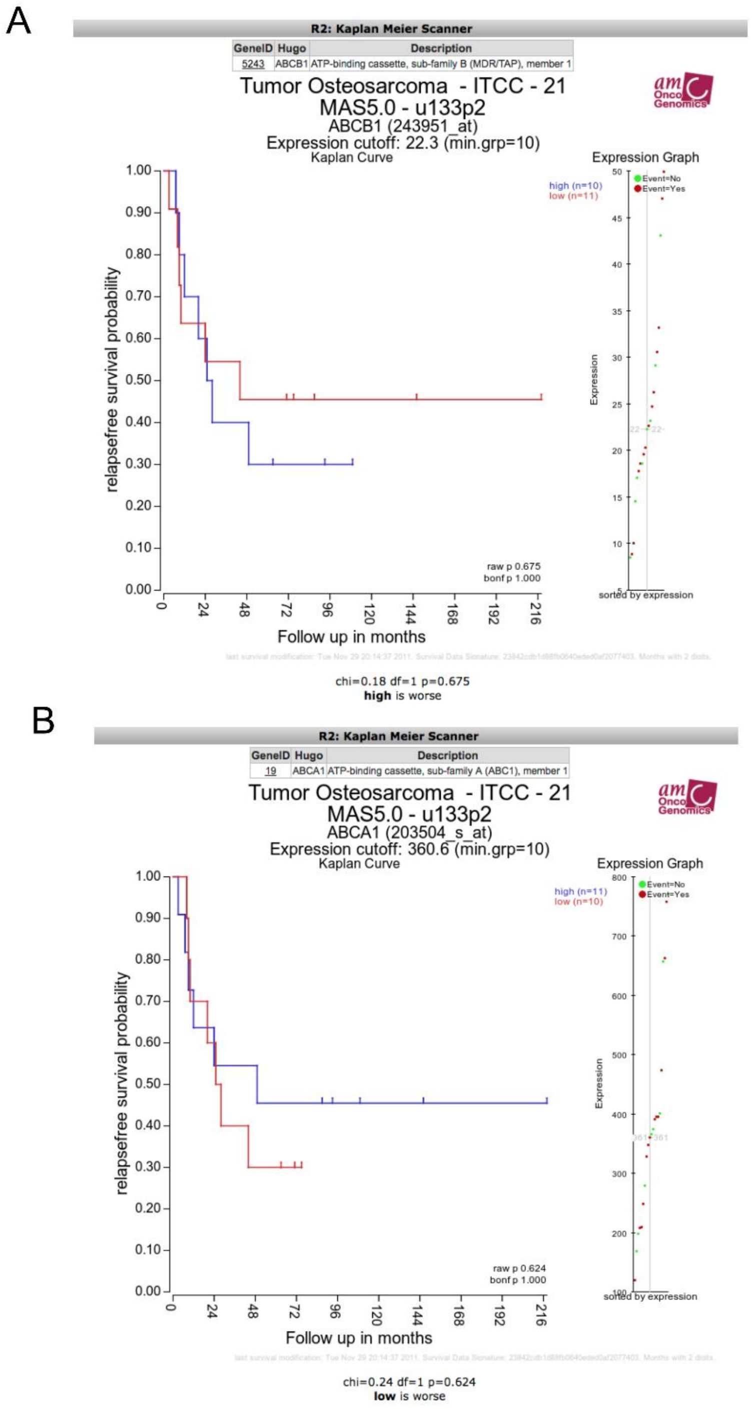

Figure S2. ABCB1 and ABCA1 mRNA expression and clinical outcome
Relapse-free survival probability of the 21 high-grade osteosarcoma patients stratified according to the median expression level of ABCB1 and ABCA1 at diagnosis. The group of high expressors (HIGH) included patients showing mRNA expression levels equal or higher to the median expression value of the whole series.

Figure S3

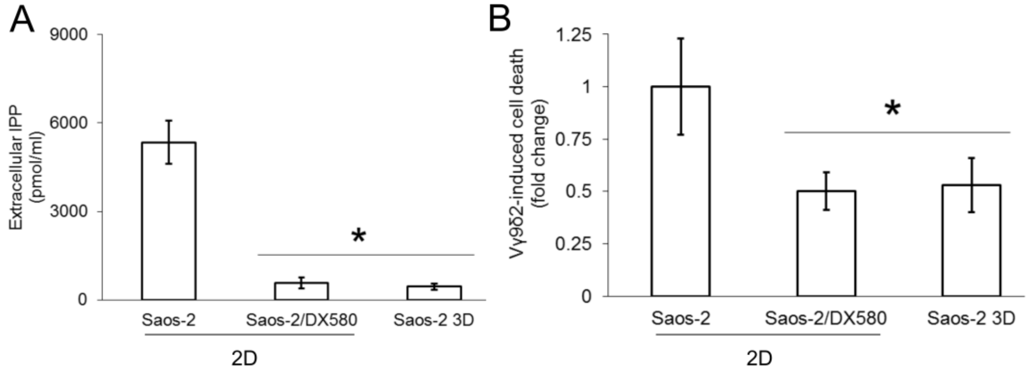

Figure S3. IPP efflux and Vγ9δ2 T-lymphocytes activity against Saos-2 cells

Saos-2 cells and Saos-2/DX580 cells were grown as 2D or 3D cultures. **A.** Cells were labelled 1 h with [^{14}C]-IPP and extensively washed. After 24 h, the [^{14}C]-IPP collected in the supernatant, considered an index of IPP efflux, was measured by liquid scintillation. Data are means±SD (n=3 independent experiments). *p<0.001 for 2D Saos-2/DX and 3D Saos-2 vs. 2D Saos-2 cells. **B.** Vγ9δ2 T-lymphocytes were cultured overnight with 2D Saos-2, 2D Saos-2/DX580 and 3D Saos-2 cells. The percentage of Annexin V/Propidium Iodide-positive cells was measured by flow cytometry, in duplicates. Data are means±SD (n=3 independent experiments). *p<0.001 for 2D Saos-2/DX580 and 3D Saos-2 vs. 2D Saos-2 cells.

Figure S4

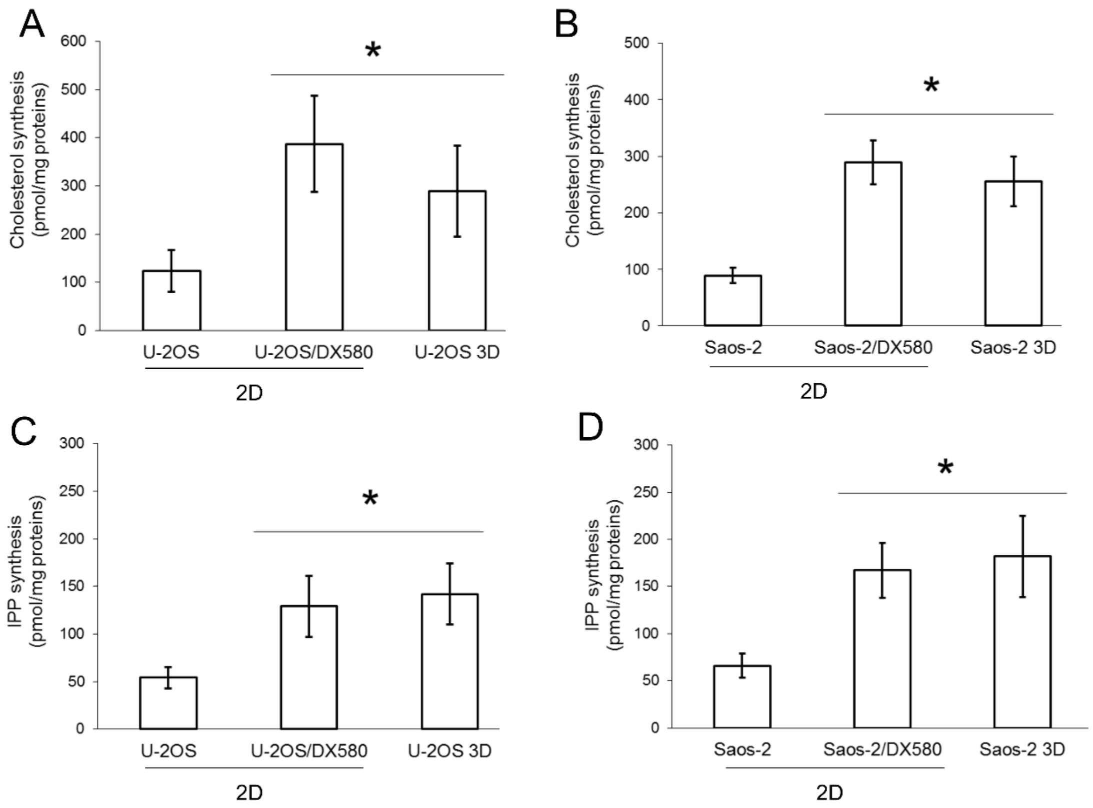

Figure S4. Endogenous synthesis of cholesterol and IPP in osteosarcoma cells
2D U-2OS, 2D U-2OS/DX580 and 3D U-2OS cells, 2D Saos-2, 2D Saos-2/DX580 and 3D Saos-2 cells were radiolabeled 24 h with [^{3}H]-acetate. **A**-**B**. The amount of [^{3}H]-cholesterol, an index of *de novo* synthesis of cholesterol, was measured by TLC separation and liquid scintillation, in duplicates. Data are means±SD (n=3 independent experiments). *p<0.01 for 2D U-2OS/DX580 and 3D U-2OS vs. 2D U-2OS cells; *p<0.001 for 2D Saos-2/DX580 and 3D Saos-2 vs. 2D Saos-2 cells. **C**-**D**. The amount of [^{3}H]-IPP, an index of *de novo* synthesis of IPP, was measured by TLC separation and liquid scintillation, in duplicates. Data are means±SD (n=3 independent experiments). *p<0.05 for 2D U-2OS/DX580 and 3D U-2OS vs. 2D U-2OS cells; *p<0.01 for 2D Saos-2/DX580 and 3D Saos-2 vs. 2D Saos-2 cells.

Figure S5

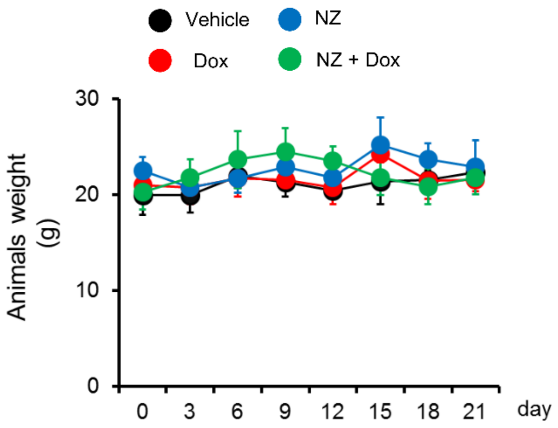

Figure S5. Tumor weight of treated animals
Hu-CD34⁺ mice were weightened at the time of randomization. The mean weigh of the whole cohort of animals was 20.95 ± 1.12 g. Mice (n=8/group) were treated as reported in Figure 5 and weighed every 3 days until the sacrifice.

Figure S6

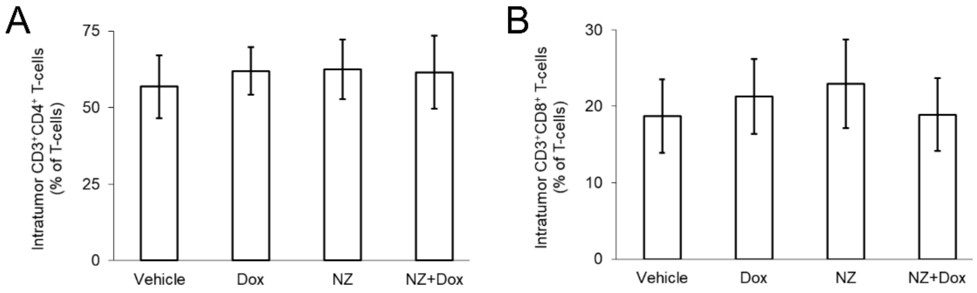

Figure S6. Immunophenotype of the intratumor lymphocytic infiltrate
1×10^6 U-2OS 3D cells were injected subcutaneously in Hu-$CD34^+$ mice. When tumor reached the volume of 50 mm^3, animals (n=8/group) were randomized and treated as indicated in Figure 5. **A-B.** Percentage of intratumor $CD3^+CD4^+$ and $CD3^+CD8^+$ T-lymphocytes vs. all $CD3^+$T-lymphocyte-infiltrating cells, measured by flow cytometry. Data are means±SD (n=8 animals).
